# Predicting Factors Affecting Postoperative Length of Stay in Patients Undergoing Coronary Artery Bypass Graft Surgery Using Machine Learning Methods: A Systematic Review

**DOI:** 10.1002/hsr2.72391

**Published:** 2026-04-22

**Authors:** Alireza Jafarkhani, Behzad Imani, Soheila Saeedi, Amir Shams

**Affiliations:** ^1^ Department of Operating Room School of Paramedicine Hamadan University of Medical Sciences Hamadan Iran; ^2^ Department of Health Information Technology School of Allied Medical Sciences Hamadan University of Medical Sciences Hamadan Iran; ^3^ Department of Cardiology Clinical Research Development Unit of Farshchian Hospital, School of Medicine Hamadan University of Medical Sciences Hamadan Iran

**Keywords:** coronary artery bypass, length of stay, machine learning, systematic review

## Abstract

**Background and Aim:**

Nowadays, coronary artery bypass graft (CABG) surgery has become a common method for treating coronary artery diseases. This surgery requires a long post‐operative length of stay (PLOS) in the hospital. The purpose of this study was to systematically review the factors affecting PLOS in patients undergoing CABG surgery using machine learning methods.

**Method:**

A comprehensive search was conducted on PubMed, Scopus, IEEE Xplore, and Web of Science, from inception until September 25, 2023. This review was performed according to the guidelines of Preferred Reporting Items for Systematic Reviews and Meta‐Analyses. All studies that investigated the factors affecting PLOS using machine learning methods in patients undergoing CABG surgery were included in the study.

**Result:**

In total, 9715 articles were identified after the removal of the duplicates. After the systematic screening, 20 studies met the inclusion criteria. The result showed there are 56 effective factors in predicting PLOS in patients undergoing CABG surgery. Of which 15 factors: age, gender, left ventricular ejection fraction, infection, Perceived Control (PC) levels, BMI, angina class, diabetes, Logistic Euro‐score, smoking, fluid balance, inotropes, low cardiac output, atrial fibrillation, and history of cerebrovascular accident are mentioned in more than one article as an affecting factors.

**Conclusion:**

This systematic review highlights the multifactorial nature of PLOS, showing that patients' postoperative length of stay is influenced by factors across pre‐, intra‐, and postoperative care.

## 1 Introduction

Nowadays, cardiovascular diseases have become one of the leading causes of death worldwide. In 2019, about 19 million people lost their lives due to cardiovascular disease [1, 2]. Coronary artery disease is considered one of the most common cardiovascular diseases, and its treatment and control of influencing factors are crucial for healthcare systems [3]. The disease's conditions determine the treatment approach, but generally, lifestyle changes, medication use, and surgical methods are among the therapeutic measures [4].

When less invasive methods are ineffective in patients, coronary artery bypass grafting (CABG) surgery becomes the most suitable treatment for coronary artery disease. Due to its positive outcomes, this surgery is widely performed worldwide, with nearly a million CABG surgeries conducted over the past decade [3, 5, 6, 7, 8]. Despite the effectiveness of CABG surgery in controlling and improving the symptoms of coronary artery disease, it is associated with complications such as chest pain and limitations in daily activities [9, 10].

CABG surgery, being an invasive procedure, requires a long postoperative length of stay (PLOS) in the hospital. Since extended hospital stays can expose patients to significant risks such as nosocomial infections or mortality, predicting PLOS in such patients is essential. It can serve as a criterion for evaluating the quality of care and surgical outcomes [5, 11]. Additionally, identifying factors that predict the duration of hospitalization can aid in the patient's clinical progress. Furthermore, since longer hospital stays incur higher costs, hospital management strives to minimize this duration [12]. Moreover, predicting patients' length of stay through accurate models cannot only assist in hospital management but also be used for prioritizing healthcare policies or enhancing treatment quality [13].

Artificial intelligence (AI) has made significant contributions to computer science and related fields in recent decades [14, 15]. Machine learning, as a branch of AI, is commonly used for analyzing large volumes of data [16]. Machine learning can predict future outcomes based on existing data when new data is presented [17]. Essentially, after learning from previously available data, machine learning uses mathematical models to make predictions [18].

Predictive analytical techniques, utilizing machine learning algorithms, can uncover hidden relationships or patterns within vast amounts of historical data [19]. Nowadays, despite the increasing volume of patient‐related data, predictive analytical techniques can be used to forecast various events, such as predicting the risk of a heart attack or the likelihood of readmission. Apart from being a promising approach for advancing clinical applications, these methods can be used to identify patients at high risk of post‐surgical complications [20, 21].

In summary, as mentioned earlier, predicting PLOS along with its influencing factors in patients undergoing CABG surgery can enhance the quality of their care. Numerous studies [22, 23, 24] have been conducted on predicting PLOS and the risk factors associated with it in these patients. However, due to the consideration of a large number of influential variables, there are differences in the results. Therefore, we aim to conduct a systematic review study to investigate the factors influencing the PLOS in patients undergoing CABG surgery, as reported in articles that utilize the machine learning method.

## 2 Materials and Methods

The present study was conducted in accordance with the Preferred Reporting Items for Systematic Reviews and Meta‐Analyses (PRISMA) guidelines proposed by Moher et al. [25] to ensure the inclusion of relevant studies.

### 2.1 Search Strategy

A systematic and comprehensive search was performed in four databases: Medline (via PubMed), Scopus, IEEE Xplore, and Web of Science, up to September 25, 2023. The search strategy included a combination of keywords and MESH terms related to machine learning and coronary artery bypass grafting surgery. A complete list of keywords is given in Table 1.

**TABLE 1:** Keywords related to searching databases.

Keywords
((“Coronary Artery Bypass”[Mesh]) OR (“Artery Bypass, Coronary”) OR (“Artery Bypasses, Coronary”) OR (“Bypasses, Coronary Artery”) OR (“Coronary Artery Bypasses”) OR (“Coronary Artery Bypass Surgery”) OR (“Bypass, Coronary Artery”) OR (“Aortocoronary Bypass”) OR (“Aortocoronary Bypasses”) OR (“Bypass, Aortocoronary”) OR (“Bypasses, Aortocoronary”) OR (“Bypass Surgery, Coronary Artery”) OR (“Coronary Artery Bypass Grafting”) OR (“Open Heart Surgery”) OR (“Open Cardiac Surgery”)) AND ((“Machine learning”) OR (“Machine Learning”[Mesh]) OR (“Deep Learning”[Mesh]) OR (“Deep Learning”) OR (“Data Mining”) OR (“Neural Network”) OR (“Genetic Algorithms”) OR (“Support Vector Machine”) OR (“Support Vector Machine”[Mesh]) OR (“Support Vector Network”) OR (“Support Vector Networks”) OR (“Supervised Machine Learning”[Mesh]) OR (“Unsupervised Machine Learning”[Mesh]) OR (“Supervised Machine Learning”) OR (“Unsupervised Machine Learning”) OR (“Random Forest”) OR (“Random Forest”[Mesh]) OR (“Random Forests”) OR (“Bayes Theorem”[Mesh]) OR (“Bayesian Network”) OR (“Artificial Neural Network”) OR (“Clustering”) OR (“Decision Tree”) OR (“Decision Trees”[Mesh]) OR (“Regression”) OR (“Bayesian”) OR (“Naive Bayes”) OR (“K‐Nearest Neighbors”) OR (“K‐Nearest Neighbor”) OR (“Genetic Algorithms”) OR (“Gradient Boosting”))

### 2.2 Selection Criteria

Articles were included if they met the following criteria:

1. Studies focused on predicting factors affecting PLOS after CABG using various machine learning methods.
2. Studies that focus solely on predicting PLOS rather than the overall hospital stay.
3. Studies that specifically predict PLOS after CABG, not for example all cardio or cardiovascular surgery, open heart surgery, or CABG combined with valve surgery.
4. Studies predicting PLOS after CABG using the on‐pump technique, excluding surgeries in which the patient did not receive on‐pump bypass or in which PLOS was compared between on‐pump and off‐pump surgeries.
5. Studies in which only the conventional method of CABG was used, excluding those performed with percutaneous, robotic, hybrid, or minimally invasive methods.
6. Studies that did not include children in their study population.

If a study had any of the following criteria, it was excluded from the study:

1. The title, abstract, or full text of the article was irrelevant to our aim.
2. Studies that were part of different chapters of books, letters to the editor, short summaries, case reports, or part of a structured review method with/without meta‐analysis.
3. Studies whose full text was not available.
4. Studies that were published in a language other than English.
5. Studies that did not mention the factors affecting PLOS and only tested a machine learning model.

### 2.3 Study Selection

After conducting an extensive and systematic search, all retrieved studies were entered into EndNote X9, a citation management software. After removing duplicate entries, two authors (AJ and SS) independently screened the titles and abstracts of all studies based on study criteria. After completing these steps and identifying the relevant articles that met our criteria, two researchers reviewed the full texts of these articles, and a final decision was made to include the relevant studies. Any discrepancies or inconsistencies were resolved through discussion and coordination with BI (supervisor of study). The full texts of the reviewed articles that did not meet the inclusion criteria were excluded from the study, and the reasons for exclusion were also checked by the research team. In the last step, the desired data was extracted from the articles included in this systematic review and entered into the Excel software.

### 2.4 Data Extraction

Data extraction was performed by the two researchers using a form designed in Excel. Any discrepancies were resolved through discussions and conversations between the researchers and the study supervisor. The extracted data include the study, first author name, the year of publication, the country, whether the article was published in a journal or presented at a conference, the name of the journal or conference, the machine learning methods used, the sample size of the study, the defined duration of the PLOS in terms of days, the source and location of data collection, whether the study focused on the postoperative length of stay in general or specifically in the intensive care unit (ICU), the accuracy of the machine learning methods, the factors influencing PLOS, whether the study was retrospective or prospective, and whether or not there were any limitations.

### 2.5 Data Analysis

Due to substantial heterogeneity in the machine learning methods used and the performance metrics reported across studies (including variation in whether accuracy, AUC, sensitivity, specificity, or predictive values were reported, as well as differences in outcome definitions and cutoff points), we did not conduct a meta‐analysis or a direct quantitative comparison of model performance. Instead, we present the results through a narrative synthesis, acknowledging that direct performance comparisons between studies are not methodologically justified.

## 3 Results

### 3.1 Results of the Literature Search

**FIGURE 1:**
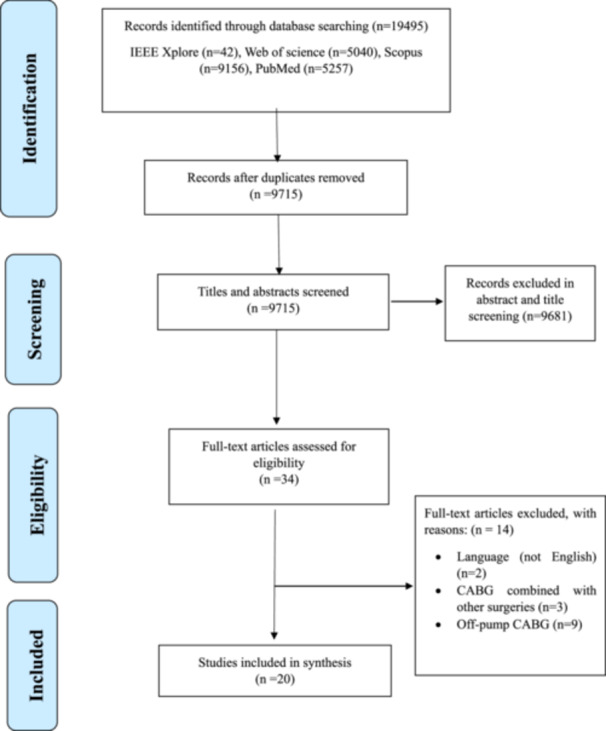
Shows the stages of identification, screening, eligibility, and inclusion of articles. The figure shows that, out of the 19,495 articles retrieved from four exploratory databases, only 20 articles were selected for the final analysis.

The results of the search in scientific databases are presented in Figure 1. A total of 19,495 articles were retrieved from the search across four databases, of which 9780 were duplicate articles. After screening the title and abstract of 9715 articles, the full text of 34 articles that seemed relevant from the view of the two screeners was investigated. After reviewing these articles, 20 articles were included in this systematic review, and 14 articles were excluded based on the inclusion and exclusion criteria.

### 3.2 General Characteristics of the Included Studies

**FIGURE 2:**
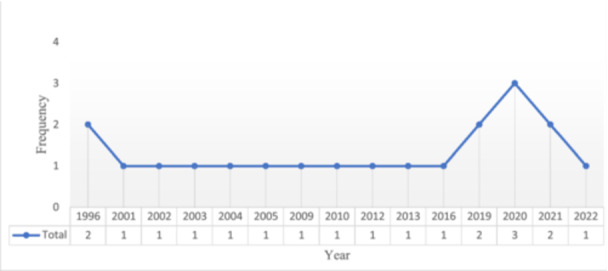
This figure illustrates the distribution of published articles per year from 1996 to 2022. Most of the related articles (*n* = 3) were published in 2020, based on the study objectives, as well as the inclusion and exclusion criteria.

The general characteristics of the 20 articles included in this study are given in Table 2. Figure 2 shows the trends of publishing related studies in PLOS prediction. The oldest article was published in 1996, and the most recent in 2022. Most articles were published with three articles in 2020 and two articles in 1996, and contrary to expectations, with the advancement of various machine learning methods, no upward trend can be seen over the years.

**TABLE 2:** General characteristics of included studies.

#	First author, year	Country	Journal/conference name	ICU or hospital PLOS prediction	Source of data	Sample size	Prospective/retrospective	PLOS	ML algorithms	Model performance
1	AbuRuz, 2022 [22]	United Arab Emirates	Nursing Practice Today	Hospital ICU	Hospital	220	Prospective	Hospital mean PLOS: 9.5 ± 9.9 days ICU mean PLOS: 6.7 ± 7.9 days	Stepwise Multiple regression	Not mention

2	Bruno, 2021 [26]	UK	British Journal of Surgery	Hospital	Hospital	2082	Retrospective	Hospital: > 6 days	Logistic regression Generalized additive model Random Decision Forest Naïve Bayes	Logistic regression: AUC = 0.71, accuracy = 0.69; Generalized additive model: AUC = 0.7, Accuracy = 0.68; Random Decision Forest: AUC = 0.7, Accuracy = 0.68; Naïve Bayes AUC = 0.63, Accuracy 0.58
3	Zarrizi, 2021 [27]	Iran	Brazilian Journal of Cardiovascular Surgery	ICU	Hospital registration system	1202	Retrospective	ICU mean PLOS: 55.27 ± 17.33 h	Cox regression	AUC = 0.697
4	Delshad, 2020 [28]	Iran	Iranian Journal of Kidney Diseases	Hospital ICU	Hospital	130	Retrospective	Hospital > 7 days ICU: > 4 days	Binary logistic regression	Not mention
5	Alshakhs, 2020 [24]	Saudi Arabia	International Journal of General Medicine	Hospital	Hospital registration system	621	Retrospective	Hospital: > 7 days	Naïve Bayes Decision Tree Random Forest Logistic Regression K‐Nearest Neighbor	Random Forest with the best fit: AUC = 0.81; F1 score = 0.82; and recall = 0.82
6	Dominici, 2020 [29]	Italy	Journal of Cardiothoracic and Vascular Anesthesia	ICU	E‐CABG registry	7352	Retrospective	ICU: ≥ 3 days	Backward stepwise multivariate logistic regression	Sensitivity = 76.0% Specificity = 62.1% ROC area = 0.686 Positive predictive value = 57.5% Negative predictive value = 79.3%
7	AbuRuz, 2019 [30]	Jordan	Risk Management and Healthcare Policy	Hospital	5 Hospital	227	Prospective	Hospital mean PLOS: 11.40 days	Stepwise multiple regression	Not mention
8	AbuRuz, 2019 [31]	Jordan	International Journal of General Medicine	Hospital	Hospital	250	Prospective	Hospital mean PLOS: 11.88 ± 10.37	Stepwise multiple regression	Not mention
9	Pustavoitau, 2016 [32]	USA	Experimental Gerontology	Hospital ICU	Hospital STS database	55	Prospective	PLOS of 7 days in the group with low levels of p16 ^INK4a^ in PBTLs, PLOS of 8.5 days in the group with high levels of p16 ^INK4a^ in PBTLs	Cox regression	Not mention
10	Oliveira, 2013 [10]	Brazil	Brazilian Journal of Cardiovascular Surgery	Hospital ICU	Hospital	104	Retrospective	ICU: > 3 days Hospital: > 7 days	Backward stepwise multivariate logistic regression	Not mention
11	Khairudin, 2012 [33]	Malaysia	ICSSBE	Hospital	Hospital	3560	Prospective	Hospital: ≥ 10	Logistic regression Decision tree	Decision tree accuracy = 65.86%, Logistic regression accuracy = 75.87%
12	Najafi, 2010 [34]	Iran	The Journal of Tehran University Heart Center	ICU	Hospital	570	Prospective	ICU: > 2 days	Stepwise multivariate logistic regression	AUC = 0.64
13	Sander, 2009 [35]	Germany	Critical Care	ICU	Hospital	59	Prospective	ICU: > 2 days	Univariate logistic regression	Not mention
14	Nakasuji, 2005 [36]	Japan	Journal of Anesthesia	ICU	Hospital	100	Retrospective	ICU: > 3 days	Multiple logistic regression	Not mention
15	Johnston, 2004 [37]	USA	The Annals of Thoracic Surgery	Hospital	Hospital	1049	Prospective	Hospital: ≥ 7 days	Forward stepwise logistic regression	ROC = 0.826
16	Hugot, 2003 [38]	France	Intensive Care Medicine	ICU	Hospital	185	Prospective	ICU: > 2 days	Forward and backward stepwise multiple logistic regression	Not mention
17	Peterson, 2002 [39]	USA	The Annals of Thoracic Surgery	Hospital	STS database	496,797	Retrospective	Hospital: > 14 days	Logistic regression Linear regression	Not mention
18	Doering, 2001 [40]	USA	Heart and Lung	ICU	Hospital	109	Prospective	ICU: > 1 day	Multivariate linear regression	Positive predictive value: 84% Negative predictive value: 42.8%
19	Michalopoulos, 1996 [41]	Greece	British Journal of Anaesthesia	Hospital ICU	Hospital	652	Prospective	> 2 days	Forward and backward stepwise multiple logistic regression	Logistic regression overall prediction accuracy = 95.1%
20	Christakis, 1996 [42]	Canada	Cardiovascular Surgery	ICU	Hospital	889	Prospective	ICU: > 3 days	Multiple logistic regression	Not mention

*Abbreviations: AUC, area under the curve; CABG, coronary artery bypass grafting; E‐CABG, European multicenter study on coronary artery bypass grafting; ICU, intensive care unit; ML, machine learning; PLOS, postoperative length of stay; ROC, receiver operating characteristic; STS, Society of Thoracic Surgeons; UK, United Kingdom; USA, United States of America; PBTLs, peripheral blood T lymphocytes; ICSSBE, International Conference on Statistics in Science, Business, and Engineering.

**FIGURE 3:**
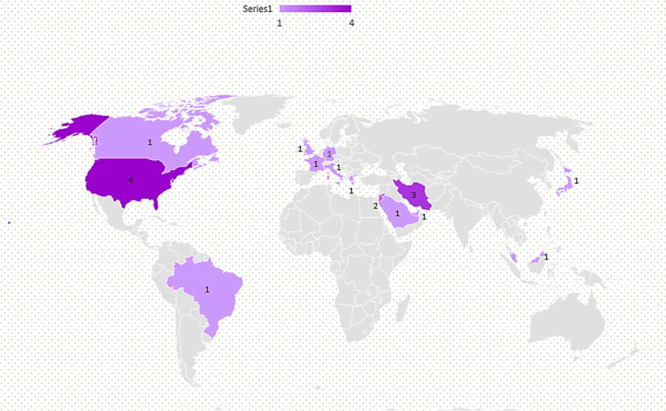
This figure shows the contribution of different countries in the publication of reviewed articles. The United States followed by Iran had the largest share of published articles.

Fourteen countries, including Brazil, Canada, France, Germany, Greece, Iran, Italy, Japan, Jordan, Malaysia, Saudi Arabia, the United Arab Emirates, the United Kingdom, and the USA, have published 20 articles related to the use of machine learning algorithms in PLOS prediction. The USA, with four articles, Iran, with three articles, and Jordan, with two articles, had published the most articles in this field, and the rest of the countries had published one article each (Figure 3).

Postoperative length of stay was defined differently across the studies we reviewed, as shown in Table 2. Some studies emphasized the postoperative intensive care unit stay (eight studies), others focused on the hospital stay (seven studies), and the remaining studies [5] reported both intensive care unit and hospital stays. Furthermore, studies differed in how PLOS was operationalized: some treated PLOS as a continuous outcome (reporting mean days), while others used binary classifications with varying cut‐off points (ranging from > 1 day to > 14 days). This heterogeneity in outcome definitions limits direct comparability between studies and should be considered when interpreting the aggregated findings.

Seventeen journals and conferences have published articles related to the use of machine learning methods in predicting factors affecting the PLOS in patients undergoing CABG. Among them, the three journals—Brazilian Journal of Cardiovascular Surgery, The Annals of Thoracic Surgery, and International Journal of General Medicine—each published two articles (Table 3).

**TABLE 3:** Journals and conferences that publish articles related to the length of stay of CABG patients.

Journals	Number
Brazilian Journal of Cardiovascular Surgery	2
The Annals of Thoracic Surgery	2
International Journal of General Medicine	2
British Journal of Anesthesia	1
Cardiovascular Surgery	1
Critical Care	1
Experimental Gerontology	1
Heart and Lung	1
Intensive Care Medicine	1
The Journal of Tehran University Heart Center	1
Iranian Journal of Kidney Diseases	1
Journal of Anesthesia	1
Journal of Cardiothoracic and Vascular Anesthesia	1
Nursing Practice Today	1
Risk Management and Healthcare Policy	1
British Journal of Surgery	1
International Conference on Statistics in Science, Business, and Engineering (ICSSBE)	1
Total	20

The data sources in most of the studies were the data recorded in the hospitals, and also the data of two registries of the “Society of Thoracic Surgeons” and “E‐CABG: The European multicenter study on coronary artery bypass grafting” was also used in the studies. The smallest sample size used in the studies was 55, and the largest was 496,797. Eight studies utilized retrospective data as input to machine learning algorithms, while 12 studies employed prospective data.

**FIGURE 4:**
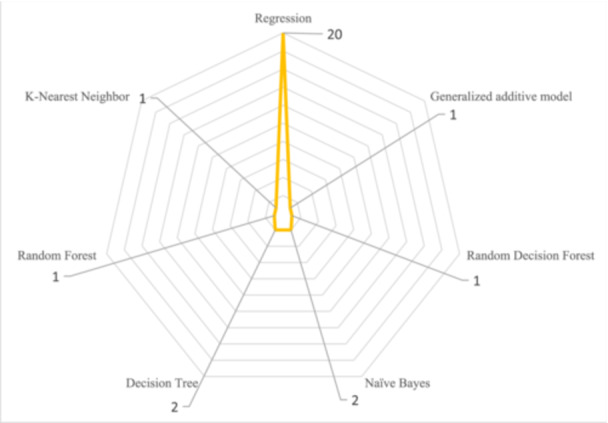
Regression methods were among the most commonly used machine learning techniques for analyzing the desired data in the reviewed articles.

The machine learning methods used to predict PLOS for patients after CABG are shown in Figure 4. Among the methods used, regression was used in all 20 studies and was the most commonly used.

Factors predicting the post‐CABG PLOS are listed in Table 4. Based on these results, age, gender, left ventricular ejection fraction, infection, perceived control (PC) levels, body mass index (BMI), angina class, diabetes, Logistic Euroscore, smoking, fluid balance, inotropes, low cardiac output, atrial fibrillation, and history of cerebrovascular accident were the predictors that mentioned as affecting factors of PLOS of patients in more than one article.

**TABLE 4:** Factors predicting length of stay in hospital after CABG.

#	Predictor	Study
1	Age	[22], [26], [24], [29], [33], [34], [36], [38], [39], [41]
2	Gender	[22], [26], [29], [30], [31], [39]
3	Left ventricular ejection fraction	[29], [10], [41]
4	Infection	[10], [33], [42]
5	PC levels	[22], [31]
6	BMI	[26], [29]
7	Angina class	[26], [29]
8	Diabetes	[10], [33]
9	Logistic Euroscore	[26], [24]
10	Smoking	[10], [42]
11	Fluid balance	[28], [40]
12	Inotropes	[41], [42]
13	Low cardiac output	[41], [42]
14	Atrial fibrillation	[27], [33]
15	History of cerebrovascular accident	[34], [42]
16	PVD	[26]
17	Having more than two chest tubes	[27]
18	Occurrence of atelectasis	[27]
19	Intra‐aortic balloon pump used	[24]
20	Height	[24]
21	Pulmonary artery systolic pressure	[24]
22	Complications during the operation	[24]
23	Poor mobility	[29]
24	Estimated glomerular filtration rate class	[29]
25	Recent potent antiplatelet use	[29]
26	NYHA class	[26]
27	Critical preoperative state	[29]
28	SYNTAX score	[29]
29	Depression scores	[30]
30	Income	[30]
31	Use of statins	[30]
32	Preoperative anxiety	[31]
33	COPD	[26]
34	Mechanical ventilation for more than 24 h	[10]
35	Hypertension	[26]
36	Chest re‐operation	[33]
37	Surgeon category	[34]
38	PDR ICG	[35]
39	Plasma levels of ASAT	[35]
40	MPAP	[36]
41	PF ratio	[36]
42	HP scale	[37]
43	The composite social risk factors score	[37]
44	Administration of catecholamines	[38]
45	Base deficit value in the first hour postoperatively	[38]
46	Comorbid illness	[39]
47	Prior surgery	[39]
48	Disease severity	[39]
49	Parsonnet score	[40]
50	Presence of arrhythmias	[40]
51	Length of intubation	[40]
52	Early hemodynamic instability	[40]
53	Aortic cross‐clamp time	[41]
54	Blood transfusions	[41]
55	Myocardial infarction	[42]
56	Bleeding	[42]

Abbreviations: ASAT, aspartate aminotransferase; BMI, body mass index; COPD, chronic obstructive pulmonary disease; HP, health perceptions; MPAP, mean pulmonary artery pressure; NYHA, New York Heart Association; PaO2/FiO2, ratio of arterial oxygen partial pressure to fractional inspired oxygen; PBTLs, peripheral blood T lymphocytes; PC, perceived control; PDR ICG, plasma‐disappearance rate of indocyanine green; PVD, peripheral vascular disease; SYNTAX, SYNergy between PCI with TAXus and cardiac surgery.

The limitations and challenges mentioned in the studies are given in Table 5. Five studies did not mention any limitations or challenges. According to the table below, it is clear that the small sample size was the most important limitation of the reviewed studies.

**TABLE 5:** Limitations of the reviewed studies.

#	Limitations	Studies
1	Small sample size	[28], [24], [32], [10], [35], [40], [42]
2	Depending on the chart review for collecting some of the information	[22], [30]
3	Failure to generalize the results of this study to other open‐heart surgeries	[24], [32]
4	Not evaluating intraoperative variables	[29], [34]
5	Selection bias	[29], [37]
6	Early discharge of patients due to the need for ICU beds.	[27]
7	Not being complete and not having the same definition of all predictors of the models of registration systems.	[27]
8	Risk of data log bias	[28]
9	Inability to accurately calculate the volume of blood lost during surgery with the usual methods	[28]
10	Missing data	[24]
11	Not including all factors that have been hypothesized to be important	[24]
12	Designing the model to predict PLoS in binary terms, that is, Above Average/Average or Below.	[24]
13	Unmeasured confounders	[29]
14	The results were dependent on the accuracy and completeness of the collected data.	[29]
15	The presented nomogram should be validated in other clinical scenarios	[29]
16	Calibration and discrimination should be externally tested	[29]
17	Prolonged length of stay in the ICU does not have a universal definition.	[29]
18	Measuring depression by a self‐reported questionnaire	[30]
19	Measuring p16 protein in the entire vascular tissue, and is unable to tell whether endothelium or smooth muscle is responsible for the reported result	[32]
20	Interpretation of findings is further complicated by the lack of a control group without CAD.	[32]
21	Focusing only on one biomarker	[32]
22	This study has low power to detect differences between groups with levels of p16INK4a mRNA in PBTLs above and below the median.	[32]
23	Evaluating the length of hospital stay as the primary outcome of interest. It is a complex outcome, which depends on multiple clinical variables, as well as administrative decisions.	[32]
24	It was a case‐control data study using previous history recorded by different observers, which may increase the heterogeneity and reduce reliability.	[10]
25	The observational nature of the study	[34]
26	Not including emergency patients	[34]
27	Inability to provide a causal relationship between ICG PDR reduction and long‐term treatment observed in the ICU	[35]
28	All patients received aprotinin as our standard antifibrinolytic therapy at that time.	[35]
29	Patients enrolled in this study were, in fact, a somewhat healthier subset of the entire concurrent Washington State CABG population.	[37]
30	Definitions for Early Discharge and Prolonged Stay cut‐points are to some degree, arbitrary.	[39]
31	Examining only PLOS, rather than the total length of stay for CABG	[39]
32	This study was not designed to understand or prescribe the “ideal length of stay” for patients.	[39]
33	Not examining the consequences of shorter or longer length of stay on patient outcomes.	[39]
34	This study was conducted at a single center	[40]
35	The number of inotropic drugs index was derived after the patient stayed in the ICU for 6 h. In this sense, it cannot be used as a preoperative prognostic indicator of duration of stay in the ICU.	[41]
36	There was a delay of a few hours in predicting the duration of stay in the ICU.	[41]

## 4 Discussion

This study was conducted to determine the parameters affecting PLOS in patients undergoing CABG surgery. It was based on articles that used machine learning methods to determine the mentioned parameters.

The study's results led to the identification of factors related to PLOS. Today, machine learning methods are widely used for various purposes in cardiac surgeries and even other specialized fields, and predicting PLOS is only a limited area of application of these methods. For example, it can refer to the study by Gokhale et al. (2023), which aimed to systematically investigate models for predicting hospital length of stay and identify related predictive factors in patients undergoing general surgery and total knee arthroplasty. The researchers of this study concluded that machine learning methods have a favorable predictive power for use in the mentioned surgeries, and this issue can promise more promising results from the use of these methods shortly [43]. We acknowledge that some studies in our systematic review used traditional regression‐based approaches rather than machine learning algorithms. Classical statistical methods, such as linear regression, are hypothesis‐driven and aim to infer relationships between variables and estimate effect sizes. These methods rely more on predefined model structures. In contrast, machine learning methods include a broader set of tools, such as random forests and artificial neural networks. These methods automatically detect patterns without strong assumptions and are more valuable in large data sets. Given the above explanations, the importance of each method, the history of the included studies, and the goals of our review, we included machine learning in this study, encompassing both traditional regression methods and newer algorithms to reflect the historical evolution of predictive modeling in clinical research. Of course, these two approaches are now considered complementary rather than incompatible.

The results of this study showed that, in general, 56 factors have an impact on PLOS in patients undergoing CABG surgery. It seems that age, gender, left ventricular ejection fraction, and infection are among the four factors that have been emphasized in more studies by researchers. Our findings regarding age parameter and ejection fraction were consistent with Almashrafi et al.'s study, as their systematic review also indicated that increased age, atrial fibrillation/arrhythmia, chronic obstructive pulmonary disease (COPD), low ejection fraction, renal failure/dysfunction, and non‐elective surgery status are likely effective factors in the length of stay in ICU after adult cardiac surgery. However, it should be noted that the general factors influencing the length of stay mentioned, which were extracted from 29 articles in Almashrafi et al.'s study, largely align with the 57 extracted factors in our study as well [12].

In general, it seems that according to the mentioned cases, increased age is effective not only in cardiac surgeries but also in other surgeries as an affecting factor in increasing the duration of PLOS [44]. It appears that the female gender as a factor plays a significant role in increasing the PLOS. Out of the six studies (Table 4) that mentioned gender as a factor, five of them considered the female gender, as reported by the researchers. In only one study, male gender was reported as an effective factor in outcome. It seems that, like age, female gender is also a risk factor in increasing the PLOS [45].

In addition, the results of the present study showed that among machine learning methods, regression was the most commonly used method in all of the reviewed studies to predict factors, while other machine learning methods were also employed. Some of the articles we studied were solely devoted to this method, while others used other methods alongside it. The result of our study was in line with the structured review study conducted by Almashrafi et al., so the result of their study on 29 articles related to heart surgeries showed that in 23 of these studies, the Multivariate logistic regression method was used as a statistical method for data analysis [12]. Of course, due to the expansion of machine learning methods, it is suggested that future studies use other machine learning methods to analyze their data.

Incorporating the findings of this systematic review into clinical practice can significantly impact the care and recovery of patients undergoing postoperative coronary artery bypass surgery. By leveraging machine learning models, hospitals can develop personalized risk assessment tools that take into account the identified factors influencing postoperative hospitalization duration. These models can help clinicians predict individual patient recovery times more accurately, enabling the development of tailored care plans and efficient resource allocation. Furthermore, by continuously updating the models with real‐world patient data, hospitals can refine and improve the accuracy of their predictions over time. This proactive approach can ultimately contribute to better patient outcomes, reduced hospital stays, and optimized resource utilization, leading to enhanced quality of care for patients undergoing coronary artery bypass surgery.

Over the 26‐year period from 1996 to 2022, our review found no consistent trend in postoperative length of hospital stay (PLOS) following coronary artery bypass graft surgery, as shown by the variability in PLOS values reported across different studies. For example, some earlier studies, such as Peterson (2002), reported hospital stays exceeding 14 days [39], while more recent studies, like Dominici (2020), also documented [29] relatively longer ICU stays over 3 days. This variation can be linked to differences in patient populations, surgical techniques, postoperative care protocols, and healthcare resources across various locations and time periods. These findings highlight the multifactorial nature of PLOS, influenced by patient demographics, comorbidities, surgical complexity, and institutional practices, which are likely more impactful than trends over time alone. Including these factors in the discussion provides a more nuanced understanding of PLOS variations and highlights the importance of individualized patient care and healthcare system factors in determining the length of stay.

One important consideration when interpreting the findings of our study is the variability in how different studies define the PLOS. For example, the distinction between ICU stay and total hospital stay is clinically important. The former is more closely related to postoperative complications and recovery, whereas the latter can reflect a wide range of social and psychological factors. This combination of factors may explain the inconsistency in identifying risk factors across our study and future studies, and may also complicate comparisons between studies. It is hoped that future studies, while using standard definitions of PLOS, will be able to provide statistical analyses suitable for meta‐analyses.

This study had some limitations and strengths. One of the strengths of our study was the comprehensive search across four key databases and the review of approximately 10,000 related articles. However, our study has some limitations. First is the exclusion of articles written in languages other than English. Another weakness of our study was the lack of inclusion of articles that investigated the factors related to the PLOS in CABG surgery with the off‐pump method.

The second limitation of the studies we reviewed was their small sample sizes (Table 5). Given that the accuracy of machine learning methods increases with larger sample sizes and that this limitation can affect study results, it is recommended that future studies, while removing this limitation, use larger sample sizes.

The Third limitation is that the current body of literature on predicting postoperative length of stay in CABG patients using machine learning is limited in its reporting and in the diversity of feature selection algorithms and hyperparameter tuning methods. Most studies relied on traditional regression‐based approaches, such as multivariate logistic regression and stepwise selection, which provide inherent variable selection but lack the complexity of advanced machine learning feature selection techniques, such as filter, wrapper, or hybrid methods. Additionally, few studies detailed the use of systematic hyperparameter optimization strategies, such as grid search or cross‐validation, which may impact model performance and generalizability. These methodological gaps highlight opportunities for future research to adopt more rigorous and transparent feature selection protocols and hyperparameter tuning to improve predictive accuracy and reliability.

The last one is methodological limitations, specifically limited attention to hyperparameter optimization, which directly affects model performance, generalizability, and scalability. In the studies reviewed, few explicitly reported using techniques such as cross‐validation to tune parameters. Although older models do not require extensive hyperparameter tuning, it is considered necessary in more complex studies. This should be taken into account in future studies.

## 5 Conclusion

This study systematically reviewed relevant articles on PLOS in patients undergoing CABG surgery using machine learning methods. The results identified 56 factors influencing PLOS in CABG surgery, with 15 having a more significant impact on increasing PLOS. Although age and gender were used as common predictors in our study, their reporting frequency should not be taken as definitive evidence of their relative importance. Instead, our findings highlight the multifactorial nature of PLOS prediction and suggest that prediction models should consider a wide range of preoperative, intraoperative, and postoperative variables. These results may assist healthcare professionals in identifying high‐risk patients and developing targeted management strategies, though future research with standardized methodologies and external validation is needed to establish more definitive predictive models.

## Author Contributions

Behzad Imani, Soheila Saeedi, Amir Shams, and Alireza Jafarkhani developed the concept for the study. Soheila Saeedi and Alireza Jafarkhani screened the articles under the supervision of Behzad Imani. Soheila Saeedi and Alireza Jafarkhani conducted the analysis and interpretation under the supervision of Behzad Imani. Finally, the manuscript was drafted by Behzad Imani, Soheila Saeedi, Amir Shams, and Alireza Jafarkhani. All authors reviewed the content and approved it.

## Ethics Statement

The study was conducted in accordance with the Declaration of Helsinki and approved by a local ethics committee in Iran, namely the Ethics Committee of the Hamadan University of Medical Sciences.

## Conflicts of Interest

The authors declare no conflicts of interest.

## Transparency Statement

The lead author Behzad Imani affirms that this manuscript is an honest, accurate, and transparent account of the study being reported; that no important aspects of the study have been omitted; and that any discrepancies from the study as planned (and, if relevant, registered) have been explained.

## Data Availability

All data generated or analyzed during this study are included in this article.
